# Metabolic Compartmentation – A System Level Property of Muscle Cells: Real Problems of Diffusion in Living Cells

**DOI:** 10.3390/ijms9050751

**Published:** 2008-05-09

**Authors:** Valdur Saks, Nathalie Beraud, Theo Wallimann

**Affiliations:** 1 Laboratory of Bioenergetics, INSERM U884, Joseph Fourier University Grenoble, France; 2 Laboratory of Bioenergetics, National Institute of Chemical and Biological Physics, Tallinn, Estonia; 3 Institute of Cell Biology, ETH Zurich Hönggerberg, HPM D24, CH-8093 Zuerich, Switzerland

**Keywords:** diffusion, metabolic (micro)compartmentation, cell structure, cytoskeleton, multienzyme complexes, channeling, regulation, Systems Biology

## Abstract

Problems of quantitative investigation of intracellular diffusion and compartmentation of metabolites are analyzed. Principal controversies in recently published analyses of these problems for the living cells are discussed. It is shown that the formal theoretical analysis of diffusion of metabolites based on Fick's equation and using fixed diffusion coefficients for diluted homogenous aqueous solutions, but applied for biological systems *in vivo* without any comparison with experimental results, may lead to misleading conclusions, which are contradictory to most biological observations. However, if the same theoretical methods are used for analysis of actual experimental data, the apparent diffusion constants obtained are orders of magnitude lower than those in diluted aqueous solutions. Thus, it can be concluded that local restrictions of diffusion of metabolites in a cell are a system-level properties caused by complex structural organization of the cells, macromolecular crowding, cytoskeletal networks and organization of metabolic pathways into multienzyme complexes and metabolons. This results in microcompartmentation of metabolites, their channeling between enzymes and in modular organization of cellular metabolic networks. The perspectives of further studies of these complex intracellular interactions in the framework of Systems Biology are discussed.

“A new scientific theory does not triumph by convincing its opponents and making them see the light, but rather because its opponents eventually die and a new generation grows up that is familiar with it”

*Max Planck Scientific autobiography and other papers, New York, 1949, pp 33–34*

## Introduction

Intensive studies of metabolic and signaling networks during more than three decades have resulted in a clear understanding of the importance of intracellularly restricted diffusion of metabolites, metabolite compartmentation and metabolite channeling for cellular metabolism, energetics, calcium signaling and signal transduction [1–3]. Indeed, numerous teams and laboratories have shown both experimentally and theoretically that the specific organization of the intracellular medium, often and wrongfully called “cytosol”, results in metabolic compartmentation, notably in muscle and brain cells [4–22]. Compartmentation, however, also means heterogeneity of intracellular diffusion [23–27]. In spite of all these developments, there are still rather numerous groups of investigators who consider the content of a cell as a homogeneous diluted aqueous medium, and this is why the philosophical, somehow skeptical commentary of Max Planck given in the epigraph of this paper seems to represent a valid and proper description of the situation. Good examples of this controversy are two recent publications on the same topic in the *Biophysical Journal*, one in May 2007 [28] and another in June 2007 [29], concerned with spatial metabolite distributions and metabolite domains. By using similar theoretical methods, the two research teams come to totally opposite conclusions. This contradiction concerns one of the most important topics in modern cellular biochemistry, that is, the influence of intracellular organization on the regulation of metabolic processes. In the most recent article of the two mentioned above, the authors, Barros and Martinez [29], arrive at the remarkable, classical, general, firm and philosophical conclusion that “the notion of cytosolic compartmentation of metabolites needs reevaluation, as it seems to be in conflict with underlying physical chemistry”. The first paper by Selivanov *et al.*, 2007 [28], however, shows exactly the opposite, that is, the real existence of metabolic compartments by modeling of spatial metabolite distribution in the cardiac sarcomere [28]. Both studies are based on the use of the same Fick's equation of diffusion [28, 29]. The principal difference is that one of articles (by Barros and Martinez) is purely theoretical and based entirely on the belief in simple diffusion in a homogenous medium [29], the second one, by Selivanov *et al.* [28] is based on a serious analysis of experimental data by quantitative methods and modeling.

Analysis of these two conflicting articles and their different historical and ideological backgrounds is most intriguing for the discussion of possible directions of development of strategies of metabolic research in the future. This is especially important because of the very rapid emergence of Systems Biology that is largely based on the application of mathematical modeling methods to systems with various complexities, in which compartmentation becomes one of the most important system – level properties, not predictable from the properties of isolated components only [27, 30–34].

## The need of experimental data for theoretical analysis

Barros and Martinez [29] consider the cell as a sphere of definite radius; inside these cells, a metabolite is produced by a single source and diffuses freely in a homogenous, isotropic medium, the “cytosol”. Thus, the cell is by definition considered as a “bag of enzymes”, these enzymes being the source of metabolites and the cell interior is a priori taken as a homogenous medium. This represents nothing less than the long - time advocated classical concept of cellular metabolism that is based entirely on mass action laws in homogenous diluted solutions, thus, on the physical chemistry of the glorious times of van't Hoff, Ostwald, Fick, Einstein – Smoluchowski and others, more than one hundred years ago. The analytical tools used by Barros and Martines [29] are restricted to the use of theory of Brownian diffusion and the turnover numbers of enzymes involved as either generators or sinks for the metabolites considered. The authors take the diffusion coefficients for metabolites determined for bulk water phase as the only realistic values, which for ATP would be equal to about 500 μm^2^/s [29]. The fact that these values for diffusion coefficients may be significantly different inside of cells with a protein concentration of approximately 200–300 mg/mL were not considered at all [29]. Thus, the possibility that physical barriers resulting from a dense cytoskeletal or mitochondrial network or from macromolecular crowding [1–27] were not taken into account at all. Taking this for granted, the authors conclude that “even under most favorable conditions that are compatible with the known physical constraints, it would be impossible that ATP pools could appear in the cytosol of a compact cell”, and then conclude that “unrealistic conditions were needed to form ATP domains” [29]. No experimental data were analyzed to verify the correctness of this model and the conclusions derived [29]. However, comparison of the same model with real experimental data was performed and published one month before in the very same *Biophysical Journal* with most remarkable results, e.g. what seemed most unrealistic to Barros and Martinez was, by analysis of experimental data by Selivanov *et al.* [28], found to be most realistic, since the experimental results showed that the apparent diffusion coefficients for ATP are locally at least three orders of magnitudes lower than those determined for the bulk water phase of the cells [28]. In the same volume of *Biophysical Journal* another article supported this conclusion: Iancu *et al.* [35] presented convincing evidence for compartmentation of cAMP in cardiac cells, with discussion of all available data on this subject, all explained by local restrictions of diffusion of cAMP and its compartmentation [35–37]. These data are in good concord with the majority of developments of cellular biochemistry and particularly cellular bioenergetics during the last three decades, which have led to a clear understanding that a cell's interior, even in “compact cells” as they are called by Barros and Martinez [29], is clearly not a homogenous solution corresponding to a “bag of enzymes” but a highly organized medium with a well defined, structured dense cytoskeleton, where macromolecular crowding and protein-protein interactions etc., play important physiological roles, e.g. by giving rise to multienzyme complexes, metabolons and other organized systems with microcompartmentation and metabolic channeling as a main mechanisms for their proper and efficient functioning, as has been described in a large number of articles (references 1–27 are only some of them), none of which was referred to by the authors of reference [29].

## Critical analysis of application of diffusion theories in cellular systems

Diffusion of metabolites in organized intracellular media has been studied for several decades with very clear results showing its restriction due to many physical factors and a multitude of parameters that are characteristic for the intracellular milieu [1–27, 38–40]. A most interesting and profound analysis has been performed by Wheatley and Agutter *et al.* [38–40], who even arrived at the rather skeptical conclusion that the classical diffusion theory could not be directly applicable for the description of intracellular transport mechanisms and that its direct and non-critical use in biology would thus represent “a relic of mechanistic materialism” [38]. Classical diffusion theory has a macroscopic aspect, relating measurable movement rates of solute molecules to concentration gradients and physical characteristics of the bulk medium by Fick's law of diffusion (derived by Fick in 1855) [41], and a microscopic aspect which explains the macroscopic phenomena in molecular terms – the Einstein-Smoluchowski model of Brownian movement, deduced independently by Einstein in 1905 [42] and Smoluchowski in 1906 [43]. The latter works led to validation of the atomic theory [44]. Fick's first equation describes the linear dependence of the rate of diffusion across the unit surface, dc/dt (or flux J, J = dc/dt) on the concentration gradient dc/dx:

(1) J=- D (dc/dx)

where the D is diffusion coefficient or diffusivity.

Both Einstein and Smoluchowski described diffusion at a microscopic level, describing its molecular mechanism – the Brownian movement [42–47]. The Einstein – Smoluchowski equation gives the relationship between diffusion coefficient, D, distance of displacement, λ, and time, t, of this displacement t:

(2) $$\text{D}=\lambda^{2}/2\text{t}$$

This equation was found for the movement in one dimension [42–47]. For two- and three-dimensional displacement of particles this equation will be D = λ^2^/4t and D = λ^2^/6t, respectively [45–47]. These equations are valid for any kind of random movement of particles, known as random walk mechanism [47].

The diffusion coefficient or diffusivity *D* described by Einstein – Smoluchowski's equation is related to the particle radius r and the viscosity of the medium η by Stokes – Einstein equation [44–47]:

(3) D= kT/6πηr

All these classical theories have been developed for weakly interacting rigid particles at sufficiently low concentrations. Agutter *et al.* have found that at least eleven conditions assumed in derivation of Fick's law and the Einstein – Smoluchovski model are not entirely met in the intracellular milieu [38–40]. The problem starts with cellular water, the intracellular mobility of which is significantly reduced, leading to partitioning of metabolites between different water phases and to changes in binding constants [38–40]. Then there is low-affinity adsorbtion of metabolites, especially if charged as ATP, to intracellular surfaces increasing the viscosity. Due to this fact, the diffusion coefficient of metabolites is decreased by a factor of (1+C/K_d_)^−1^ where C is concentration of binding sites and K_d_ is dissociation constant of solute from these complexes [40]. Furthermore, macromolecular crowding and cytoskeletal structures create barriers which increase the effective path-length of diffusion, and again, the diffusion coefficient is decreased by Δλ^2^ where Δλ is relative increase in path attributable to the barriers [38–40]. Finally, the movements of individual molecules become co-ordinated and vectorially directed due to organization of enzymes into multi-enzyme complexes, and the randomness of molecular events may be lost [40, 48–49]. Agutter *et al.* even recommend not to use “diffusivities” or “diffusion coefficients” for biological systems but to use some terms of the type of “empirical transport coefficient” when the Fick's equation is formally applied for intracellular processes [40]. In our analysis of local restrictions of the diffusion of ATP and ADP in the cells, we have used the term “apparent diffusion coefficient, *D*^*app*^ “, *D*^*app*^ = *DFxD*_*0*_, where *D*_*o*_ is the diffusion coefficient in bulk water phase and DF is a diffusion factor accounting for all intracellular mechanisms locally restricting particles movement [23, 25, 26, 50]. The diffusion coefficients calculated by Selivanov from analysis of experimental data [28, 51, 52] have the same meaning of apparent constants. Both our and Selivanov's results show that *DF* value in some areas of cells, for example in myofibrils, close to sarcolemma and mitochondrial outer membrane may be in the range of 10^−2^–10^−5^ [23, 28, 50–52].

## Structural basis for diffusion restrictions and compartimentation

The results of these local diffusion restrictions are microcompartmentation of metabolites and their channeling within organized multi-enzyme complexes which need to be accounted for to explain many biological phenomena. Indeed, none of the important observations in cellular bioenergetics could be explained by a paradigm describing a viable cell as a “mixed bag of enzymes” with homogenous metabolite distribution, since this simplistic theory excludes any possibility of metabolic regulation of cellular functions.

### a) Macromolecular crowding, heterogeneity of diffusion, compartmentation and vectorial metabolism

The first phenomenon to be taken into account in all cells is macromolecular crowding: the high concentrations of macromolecules in the cells [4–9] decrease the volume available for free diffusion of substrates and accordingly make it difficult to use correctly those enzyme kinetics calculations and equations that were mostly worked out based on enzymatic studies carried out in dilute solutions with isolated enzymes [8–10]. Cytoplasmic protein concentration may be as high as 200 to 300 mg/mL, which corresponds to a volume fraction of about 20–30% of intracellular medium occupied by these proteins [4, 5]. In mitochondria, the high density of enzymes and other proteins constitutes more than 60% of the matrix volume [53]. Such a specific macromolecular architecture of the cells can be revealed and experimentally studied by cryoelectron tomography [54]. The effects of macromolecular crowding on diffusion in virtual cytoplasm were recently analyzed by Ridgway *et al.*, who showed that only due to this important characteristics of intracellular medium, the apparent diffusion coefficients may be decreased by order of magnitude depending upon the size of the diffusing particles and occupied volume fraction [55].

At first sight, this macromolecular crowding should cause a real chaos by making intracellular communication by diffusion of reaction intermediates very difficult. This chaos and related problems are well described by Noble in his recent book [30]. In reality, however, macromolecular crowding gives rise to new mechanisms based on specific protein – protein interactions: microcompartmentation, metabolic channeling and functional coupling, and in this way to a fine organization and regulation of metabolism. The origin of this order, the program and source of these specific interactions and cellular organization are still a mystery, hidden in undiscovered genetic laws, including feedback control of gene expression, and in rules governing system level properties of cellular systems [30–32]. Their elucidation will be the main challenge for cell sciences in the post–genomic era [30–34].

### b) Heterogeneity of intracellular diffusion, metabolic channeling

Many new experimental techniques have been developed to study the molecular networks formed by protein–protein interactions [56]. In the cells, the high protein density predominantly determines also the major characteristics of cellular environment such as diffusion in heterogeneous compartments [11, 13, 14, 57, 58]. There are distinct barriers to diffusion of solutes within the cells: binding and crowding. Whereas molecular crowding and sieving restrict the mobility of very large solutes, binding can severely restrict the mobility of even smaller solutes ([14] and references therein). This also explains the heterogeneity of the diffusion behavior of ATP in cells. Studies, using pulsed-gradient ^31^P-NMR, showed that the diffusions of ATP and phosphocreatine both are anisotropic in muscle cells [57, 58]. Recent mathematical modelling of the decreased affinity of mitochondria for exogenous ADP *in situ* in permeabilized cardiac cells also showed that ADP or ATP diffusion in cells is heterogeneous and that the apparent diffusion coefficient for ADP (and ATP) may be locally decreased (diffusion locally restricted) by an order, or even several orders of magnitude [50]. A similar limited diffusion of ATP in the subsarcolemmal area in cardiac cells was proposed by the Terzic and Dzeja group [51, 52]. In sea urchin sperm cells, it was shown that diffusion of ATP from the mitochondrion to the sperm tail and much more so of ADP from the sperm tail back to the mitochondrion were severely restricted and orders of magnitude slower than that of phosphocreatine and creatine, respectively [59]. There is also firm experimental evidence for compartmentalization of ATP in cardiomyocytes (see next section).

Due to molecular crowding and hindered diffusion cells need to compartmentalize metabolic pathways in order to overcome diffusive barriers. Biochemical reactions can successfully proceed and even be facilitated by metabolic channeling of intermediates due to structural organization of enzyme systems into organized multienzyme complexes. Metabolite channeling directly transfers the intermediate from one enzyme to an adjacent enzyme without the need of free aqueous-phase diffusion [1–3, 6, 13, 48, 49, 60]. This property was suggested to be a unique catalytic behavior of enzyme complexes due to their specific structural organization [17]. Gaertner reported that “physically associated multienzyme systems (enzyme clusters) have the potential of expressing unique catalytic properties in contrast to their non-associated counterparts” [17]. It is quite clear that the enhanced probability for intermediates to be transfered from one active site to the other by sequential enzymes requires stable or transient interactions between the relevant enzymes. Enzymes are able to associate physically in non-dissociable, static multienzyme complexes, which are not random associations but an assembly of sequentially related enzymes.

### c) Compartmentation phenomenon and vectorial metabolism

Thus, the principal ways and mechanisms of organization of cell metabolism are macro- and microcompartmentation, metabolic channeling and functional coupling. By definition, the term compartmentation is usually related to the existence of intracellular *macrocompartments* – subcellular regions which are large relative to the molecular dimension, and *microcompartments* which are of the order of the size of metabolites. Compartment means “subcellular region of biochemical reactions kinetically isolated from the rest of cellular processes” [61, 62]. Macrocomparments are easy to understand, and they can be visualized by electron or confocal microscopy. They are compartments inside organelles such as mitochondria or lysosomes, and are insulated from the cytoplasm by membrane envelopes. The concept of microcompartmentation is more mysterious, since these microcompartments are not usually visible by electron microscopy. However, now it is becoming clear that microcompartments related to multienzyme complexes and metabolic channeling, as described above, are the principal basis of organization and compartmentation of cellular metabolism. They are formed by specific protein-protein interactions within multienzyme complexes due to the macromolecular crowding. For example, anchoring of the glycolytic [63–67] and other enzymes to the cytoskeleton or to membrane channels and transporters [68] has been demonstrated. Investigations carried out in Clegg's and Deutscher's laboratories have shown that mammalian cells behave as highly organized macromolecular assemblies dependent on the cytoskeleton [20, 21]. Multienzyme complexes may be of different size and even include whole metabolic pathways – then they are called metabolons, according to the terminology introduced by Srere in 1985 [22]. Thus, there are glycolytic [64–67], Krebs cycle [69] and many other metabolons [70, 71]. New techniques such as FRET have been developed to study and visualize such micro-compartments, of which a good example is the study of microcompartmentation of cyclic AMP [36, 37]. Microcompartmentation is sometimes taken to be synonymous of metabolic channeling. However, metabolic channeling of the reaction intermediate between two enzymes (or a transporter and an enzyme) may occur *via* microcompartments or by direct transfer [48, 49, 60]. In both cases, it results in functional coupling (see below). Importantly, microcompartmentation may be of dynamic nature, and this may finally result in co-existence of a whole set of organized metabolic networks [62].

Interestingly enough, there is an exciting hypothesis that these phenomena, in particular metabolic channeling, are even older than life itself and related to its origin. Edwards and others (see [72] for a review) have put forward the hypothesis according to which on the prebiotic earth, when no enzyme or metabolic complexes were initially present, archetypal catalytic complexes were formed at the mineral surface (for example, iron sulfide minerals) and biomolecules and catalysts would have been formed at specific sites relative to these complexes [72]. The evolution of metabolic pathways in this case would have been dictated by the relative positions of substrates and catalysts, where only closely juxtaposed species would have been allowed to interact [72]. Thus, the cell as a “bag of enzymes” probably never existed. It is now becoming clear that the living cells are much more complicated, and much better organized than thought before [15, 16].

In the case of metabolic channeling of species *via* microcompartments with only a small number of molecules, the validity of introducing chemical potentials is questionable [49], whereas for macrocompartments and larger microdomains this classical theory remains useful. In the first case, application of thermodynamic activities instead of concentrations [7–9], and stochastic kinetics based on probabilities of different states of enzymes will be essential [49, 73].

An important consequence of the organization of the enzymes into multienzyme complexes is vectorial metabolism and ligand conduction, a general principle proposed by Peter Mitchell after extensive enzymological studies and detailed characterization of mitochondrial proteins. One important example is the chemiosmotic coupling of energetic processes through the “protonic current” [74, 75]. It brought together “transport and metabolism into one and the same chemiosmotic molecular level - biochemical process catalyzed by group-conducting or conformationally mobile group-translocating enzyme system” [74]. In his latest reviews, Mitchell encouraged a wider use of the chemiosmotic principle and the biochemical concept of specific ligand conduction in explaining organization and operation of metabolic and transport processes within the cell [74]. Today this idea receives increased attention and is certainly another important insight of Mitchell to the understanding of cellular energy conversion processes.

## Compartmentation of ATP and ADP in cardiac and brain cells – a system level property

Cellular bioenergetics of cardiac cells provides plenty of evidence for compartmentation of metabolites, most clearly – of adenine nucleotides -, some of them are described below. It was discovered by Neely *et al.* [76, 77] and Gudbjrnason *et al.* [78] some thirty years ago that under ischemic or hypoxic conditions, cardiac contraction stops in the presence of about 80–90% of original ATP, but when most of the phosphocreatine is exhausted. Recent studies by the Neubauer group showed that in patients with dilated cardiomyopathy, the high mortality rate is related to a decreased PCr/ATP ratio below 1.6 meaning again that even if global ATP content in the cells stays high, the level of phosphocreatine is critically important, not only for maintaining global ATP, but much more so local ATP concentrations [79]. If there were a homogenous intracellular medium with unrestricted diffusion, why then would this homogenous pool of ATP not be used to support the cellular functions [76–80]? Some time ago, it was experimentally shown that anomalies in diffusion behavior and equilibration of ATP and other metabolites, contrary to such as expected from Brownian diffusion, can indeed be directly measured *in vivo* [81, 82]. These data explain both the reasons for realistic diffusion restrictions inside the cells and metabolic compartmentation, within a combined framework of experimental and theoretical research. An overwhelming body of experimental evidence shows the existence of distinct ATP pools (compartments) in mitochondria, as well as at the sites of ATP utilization that are connected by phosphotransfer networks, notably via PCr-creatine kinase pathway (reviewed in [24–27, 59, 83–90]). These networks function to bypassing and overcoming of the local restrictions of ATP or ADP diffusion and thus perform the important tasks of both energy supply and metabolic feedback regulation of respiration. When PCr decreases in the cell, local ATP pools are rapidly exhausted by ATPases with all pathogenetic consequences (cessation of contraction) [24, 25, 27, 77, 89, 90]. Paul's laboratory in Cincinnati has demonstrated the compartmentation of oxidative and glycolytic metabolic systems, as well as PCr-CK pathway, even in smooth muscle cells that are rather small compared to skeletal muscle cells [91]. As another example, excitation – contraction coupling which controls cardiac contraction and bioenergetics is strictly dependent on the action potential duration and plasma membrane (sarcolemmal) repolarisation via K-ATP channels [92]. These channels were discovered by Noma, who found a very high affinity of these channels for ATP, the apparent Km (ATP) being about 0.1 mM [93]. ATP inhibits the opening of this channel. Nevetherless, in cells *in vivo*, these channels are open after a significant decrease of the PCr content in the presence of bulk ATP that is still high in millimolar concentration [51, 52]. Again, combined experimental and theoretical studies of the opening of the K-ATP channels by Abraham *et al.* [51] showed that in the subsarcolemmal area, the diffusion of ATP is strongly restricted and the apparent diffusion coefficient for this nucleotide calculated from Fick's equation may be decreased *in vivo* in this cellular microcompartment by five orders! of magnitude, meaning that the K-ATP channel is not seeing bulk ATP but rather a very local ATP compartment that can be quite different in concentration from bulk ATP. And again “unrealistic” becomes realistic, if theoretical calculations are carefully checked by experiments! Numerous experimental and theoretical studies concerning the regulation of respiration in permeabilized cardiomyocytes by exogenous ADP have revealed the local restrictions of ADP diffusion in cells and again support the concept of compartmentation of adenine nucleotides between multiple subcellular pools, most probably microcompartments [3, 23–27, 34, 79, 84].

Remarkably, in the heart the intracellular energy transfer networks are structurally organized in the intracellular medium where macromolecules and organelles, surrounding a regular mitochondrial lattice, are involved in multiple structural and functional interactions [3, 25–27, 34]. These complexes were called ‘intracellular energetic units”, ICEUs, and taken to represent the basic pattern of organization of muscle energy metabolism [25, 26]. There are no physical barriers between ICEUs, each mitochondria (or several adjacent mitochondria) can be taken to be in the centre of its own ICEU. This concept describes the organized functional connections of mitochondria with their neighbours. ICEUs are analogous to calcium release units, (CRUs), structurally organized sites of Ca^2+^ microdomains (Ca^2+^ sparks) which form a discrete, stochastic system of intracellular calcium signaling in cardiac cells [94]. The structural organization of ICEUs results in local confinement of adenine nucleotides and Cr/PCr couple in discrete dynamic energetic circuits between actomyosin and membrane ATPases and mitochondrial ATPsynthase [24–27, 34, 84–87]. These phosphotransfer networks are necessary for effective energy transfer from mitochondria to all sites of energy utilization under conditions when the diffusion of ATP and ADP may be locally restricted due to structural organization of the cardiac cells [23–28, 50–52, 83–90]. Important studies of mitochondrial membrane topology by Mannella's and Perkins’ laboratories [95, 96] using electron tomography have revealed narrow tubular junctions between intracristal compartments and intermembrane space which represent first diffusion barriers for adenine nucleotides. Next are the restrictions of the permeability of the outer mitochondrial membrane due to interactions of voltage-dependent anion channels (VDAC) of this membrane with cytoskeletal components and diffusion restrictions within ICEUs [23, 61]. To overcome all these restrictions, mitochondrial ATP is transported across the inner membrane by adenine nucleotide translocase (ANT) directly to mitochondrial creatine kinase (MtCK) in exchange to ADP produced by MtCK [3, 24–27]. Due to effective functional coupling between ANT and MtCK, ATP and ADP are continuously recycling within mitochondria, energy is carried out into cytoplasm by phosphocreatine molecules and respiration is controlled to significant extent both by the MtCK reaction and inorganic phosphate fluxes between ATPases and mitochondria [23–28, 50–52, 83–90]. This sequence of events has been experimentally confirmed with application of new methodologies of investigations of *in vivo* kinetics of the energy transfer by which high-energy phosphoryl fluxes through creatine kinase, adenylate kinase and glycolytic phosphotransfer, captured with ^18^O-assisted ^31^P-NMR, were shown to tightly correlate with the performance of the myocardium under various conditions of load [34, 97, 98], implicating phosphotransfer reactions as indispensable routes that direct flow of high energy phosphoryls between cellular ATPases and the ATP production machinery in mitochondria [34]. Very similar results were obtained by ^31^P-NMR saturation or inversion transfer methods [99, 100]. Most remarkably, these phosphotransfer networks ensure effective metabolic feedback signalling to mitochondria under physiological conditions of metabolic stability when heart function and respiration is governed by the classical Frank-Starling law of the heart [34]. The most recent publications show that the phosphocreatine (PCr) – creatine kinase (CK) system is equally important in skeletal muscle function and development: it is the PCr-CK system which sustains localized ATP-dependent reactions during actin polymerization in myoplast fusion to form myotubes during myogenesis [101]. All these results add new insight into the functioning of the PCr-CK system in muscle cells, showing its new role in energy supply for cytoskeletal remodelling. By controlling the rate of regeneration of ATP in local microcompartments, the PCr-CK system helps to shape the muscle cells, to keep them healthy and alive [101]. Similar discrete microdomains in cardiomyocytes have been shown for cAMP in the range of approximately 1 μm exhibiting high local concentrations [35–37]. This concept is supported by recent work published by Weiss *et al.* [102]. These authors presented a holistic view of cardiovascular metabolism, considering it from the perspective of physical network, in which various metabolic modules are spatially distributed throughout the interior of the cell to optimize ATP delivery to specific ATPases [102]. In addition to mitochondrial module (which is represented ICEUs), the authors considered also a module consisting of glycolytic enzyme complexes serving for energy channeling to molecular complexes in sarcolemma and sarcoplasmic reticulum, and modules of calcium cycling [102]. These modules were further analyzed from the abstract perspective of fundamental concepts in network theory [103, 104] and dynamic perspective of interactions between modules [102]. The authors emphasized that understanding the nature of these interactions within hiercharchical modular structures is a main challenge of research of cardiac metabolism to gain deeper understanding of possible mechanisms of cardioprotection [102].

Some 10 years ago, based on measurements, utilizing a new technology that made it possible to directly measure by saturation transfer ^31^P-NMR global creatine kinase flux in muscle *in vivo*, some laboratories [105, 106] came to conclusions similar to those in [29] saying that “the CK chemical reaction kinetics in the intact cell is adequately described in terms of a well-mixed solution of enzyme, substrate and products”, as also published in *Biophysical Journal* [105], and that “the thermodynamic characteristics of the cytosol can be predicted as if these metabolites were freely mixing in solution” [106]. This simplistic view has been refuted, among others mostly by arguments based on the specific subcellular localization of creatine kinase isoenzymes at distinc compartments which cannot be resolved by global bulk NMR methods [107] and on anomalous ^31^P-NMR flux behaviour of the creatine kinase system in creatine kinase knock-out mice or such with graded expression of creatine kinase [107, 108], as discussed in detail [109]. Now, it seems that similar views are resurrected again by some members of the research community involved in metabolic modelling approaches [29].

However, modern biophysical chemistry can no longer be restricted to classical theories of diluted homogenous solutions, as misleadingly taken by Barros and Martinez [29]. The second important conclusion of these considerations is that purely theoretical constructs have a very limited (mostly only virtual, as some good computer games) value if not checked, verified and corrected by inputs from actual experimental results.

What modern biophysical chemistry needs is to critically reevaluate of use of the principles of classical physical chemistry that have been worked out for diluted aqueous solutions and to adapt these concepts to the *in vivo* situation for cells that possess an extensive myofilament lattice, as muscle cells, or a dense cytoskeletal an mitochondrial network and other diffuson restrictions inferred by intracellular organization or macromolecular crowding. Moreover, biological sciences witness now a radical change of paradigms. Reductionism that used to be the philosophical basis of biochemistry and molecular biology, when everything from genes to proteins and organelles was studied in their isolated state, are superseded by Systems Biology, a holistic view that favours the study of integrated systems at all levels: cellular, organ, organism, and population, accepting that the physiological whole is greater than the sum of its parts [3, 30–34]. Reductionism was justified in the initial stages of biological research giving a wealth of information on system components. The aim of Systems Biology is to put them together and analyze them in interaction, to understand the principles of functioning of the whole. At the cellular level, it is becoming clear that most of biological characteristics arise from complex interactions between the cell's numerous constituents, and based on protein-protein interactions, cellular metabolism is likely to be carried out in a highly modular manner within hierarchically organized networks [3, 102]. The situation is definitely contrary to that described by Barros and Martinez [29], who contend that “the cytosol behaves as a well-mixed, homogeneous compartment”. The *in vivo* situation is much more realistically described and well illustrated by Selivanov *et al.* [28]. Clearly, his model is also a simplified one, a first approximation to complex phenomena of microcompartmentation and metabolic channeling, and although critical evaluation of the Selivanov paper [28] also reveals some weak points and shortcomings, due to assumptions for modelling that are only indirectly derived from experimental data. The fact that compartmentation and interactions between myofibrillar components are taken into account in his model calculations makes this approach a more realistic one, which is likely to better reflect the *in vivo* situation. Apparent diffusion coefficients *D*^*app*^ and diffusion factors *DF* (see above), as well as compartmentation, are system level properties not predictable from isolated components but arising from their interactions [3, 30–34]. The real problems and challenges for further studies are both to measure local concentrations of metabolites, including those of ATP in different cellular microcompartments and its metabolic channelling within microdomains (local fluxes), as well as to fully understand the nature of these restrictions of diffusion upon which intracellular compartmentation is based. This difficult work is necessary for reasonable computer modeling of the hierarchical modules of metabolic networks as a part of Systems Biology [32, 33]. High throughput methods have now been developed to determine thousands of protein-protein interactions within cells at a given metabolic state, and it may soon be possible to also define changes in protein-protein interactions, for example, as a consequence of cell stimulation and cell signaling and thus being able to study the dynamics of metabolons. This is possible by cross-linking proteins via isotopically tagged chemical cross-linkers in cells *in situ* and, upon proteolytic digestion and determintation of the isotopically labeled peptides by mass spectrometry, sift through large protein sequence data bases to identify the cross-linked peptides and attribute them to the original partner proteins that at the time and conditions of chemical cross-linking did interact with each other [110].
